# Identification of Zoophilic Dermatophytes Using MALDI-TOF Mass Spectrometry

**DOI:** 10.3389/fcimb.2021.631681

**Published:** 2021-04-28

**Authors:** Christina-Marie Baumbach, Stefanie Müller, Maximilian Reuschel, Silke Uhrlaß, Pietro Nenoff, Christoph Georg Baums, Wieland Schrödl

**Affiliations:** ^1^ Institute of Bacteriology and Mycology, Centre of Infectious Diseases, Faculty of Veterinary Medicine, Leipzig University, Leipzig, Germany; ^2^ Clinic for Small Mammals, Reptiles and Birds, University of Veterinary Medicine Hannover, Foundation, Hannover, Germany; ^3^ Laboratory for Medical Microbiology, Mölbis, Germany

**Keywords:** ****dermatophytoses, zoonoses, hedgehog, zoophilic, geophilic, *Trichophyton*, *Microsporum*, *Trichophyton erinacei*

## Abstract

Dermatophytoses**** represent a major health burden in animals and man. Zoophilic dermatophytes usually show a high specificity to their original animal host but a zoonotic transmission is increasingly recorded. In humans, these infections elicit highly inflammatory skin lesions requiring prolonged therapy even in the immunocompetent patient. The correct identification of the causative agent is often crucial to initiate a targeted and effective therapy. To that end, matrix assisted laser desorption ionization time-of-flight mass spectrometry (MALDI-TOF MS) represents a promising tool. The objective of this study was to evaluate the reliability of species identification of zoophilic dermatophytes using MALDI-TOF MS. The investigation of isolates from veterinary clinical samples suspicious of dermatophytoses suggests a good MALDI-TOF MS based identification of the most common zoophilic dermatophyte *Microsporum canis. Trichophyton (T.)* spp. usually achieved scores only around the cutoff value for secure species identification because of a small number of reference spectra. Moreover, these results need to be interpreted with caution due to the close taxonomic relationship of dermatophytes being reflected in very similar spectra. In our study, the analysis of 50 clinical samples of hedgehogs revealed no correct identification using the provided databases, nor for zoophilic neither for geophilic causative agents. After DNA sequencing, adaptation of sample processing and an individual extension of the in-house database, acceptable identification scores were achieved (*T. erinacei* and *Arthroderma* spp., respectively). A score-oriented distance dendrogram revealed clustering of geophilic isolates of four different species of the genus *Arthroderma* and underlined the close relationship of the important zoophilic agents *T. erinacei, T. verrucosum* and *T. benhamiae* by forming a subclade within a larger cluster including different dermatophytes. Taken together, MALDI-TOF MS proofed suitable for the identification of zoophilic dermatophytes provided fresh cultures are used and the reference library was previously extended with spectra of laboratory-relevant species. Performing independent molecular methods, such as sequencing, is strongly recommended to substantiate the findings from morphologic and MALDI-TOF MS analyses, especially for uncommon causative agents.

## Introduction

Dermatophytoses are common worldwide and represent a growing health concern for human patients, companion animals and livestock alike (Chermette et al., 2008; Havlickova et al., 2008). These superficial infections of skin and its appendages (hair, nail, fur, spines, hoofs, claws etc.) are often caused by dermatophytes, which are able to invade the aforementioned host structures and digest the tough, fibrous proteins forming their structural framework. They belong to three main genera, i.e. *Epidermophyton (E.)*, *Microsporum (M.)* and *Trichophyton (T.)*, and are categorized as anthropophilic, zoophilic and geophilic according to their preferential habitat and evolutionary adaptation to humans, animals and soil, respectively (Weitzman and Summerbell, 1995). About 40 species are of clinical relevance in human and veterinary medicine nowadays (Ferguson and Fuller, 2017).

Zoophilic dermatophytes are animal pathogens that often exhibit a strong host specificity but also a notable zoonotic potential. Human infections with zoophiles (and also geophiles) are highly inflammatory, contagious and the patients frequently need systemic and long-lasting treatments (Weitzman and Summerbell, 1995; Havlickova et al., 2008). Especially children and adolescents are affected due to close contact to household pets, e.g. cats and guinea pigs, that are often asymptomatic carriers (Chermette et al., 2008; Nenoff et al., 2014a).

Routine diagnostic identification of dermatophytosis-causing agents continues to be mostly accomplished by macroscopic and microscopic examination of mycological cultures, i.e. mycelia, fruiting bodies and characteristic conidia. This approach is time-consuming and requires expert knowledge due to remarkable morphological similarities between the different species (Nenoff et al., 2013). On the other hand, DNA sequencing, which is considered the “gold standard” for species identification, is laborious and resource-expensive (Nenoff et al., 2013; Heireman et al., 2020). Moreover, nucleic acid sequence comparison relies on public, not necessarily validated databases that also need to be interpreted cautiously because of non-standardized preparation methods and the ongoing renaming and reclassification of dermatophytes (Abarca et al., 2017).

On the contrary, the identification of microorganisms based on matrix assisted laser desorption ionization time-of-flight mass spectrometry (MALDI-TOF MS), particularly of bacteria and yeasts, is considered simple, fast and reliable. However, for clinically relevant filamentous fungi, and especially dermatophytes, the thus-far established sample preparation methods need extensive adaptations since growth requirements and sample processing are more elaborate in these species (van Veen et al., 2010; Carbonnelle et al., 2011; Juiz et al., 2012; Biswas and Rolain, 2013; L’Ollivier and Ranque, 2017).

Here, we examined the reliability of MALDI-TOF MS for the identification of closely related zoophilic dermatophytes. Furthermore, we compared the spectra obtained from growth in two different conditions, i.e. liquid broth vs. solid agar media. Finally, we describe a cohort of samples from our veterinary clinical practice where the extension of the reference database was crucial to identify the uncommon causative agents involved.

## Material and Methods

### Sampling and Fungal Culture

**Figure 1:**
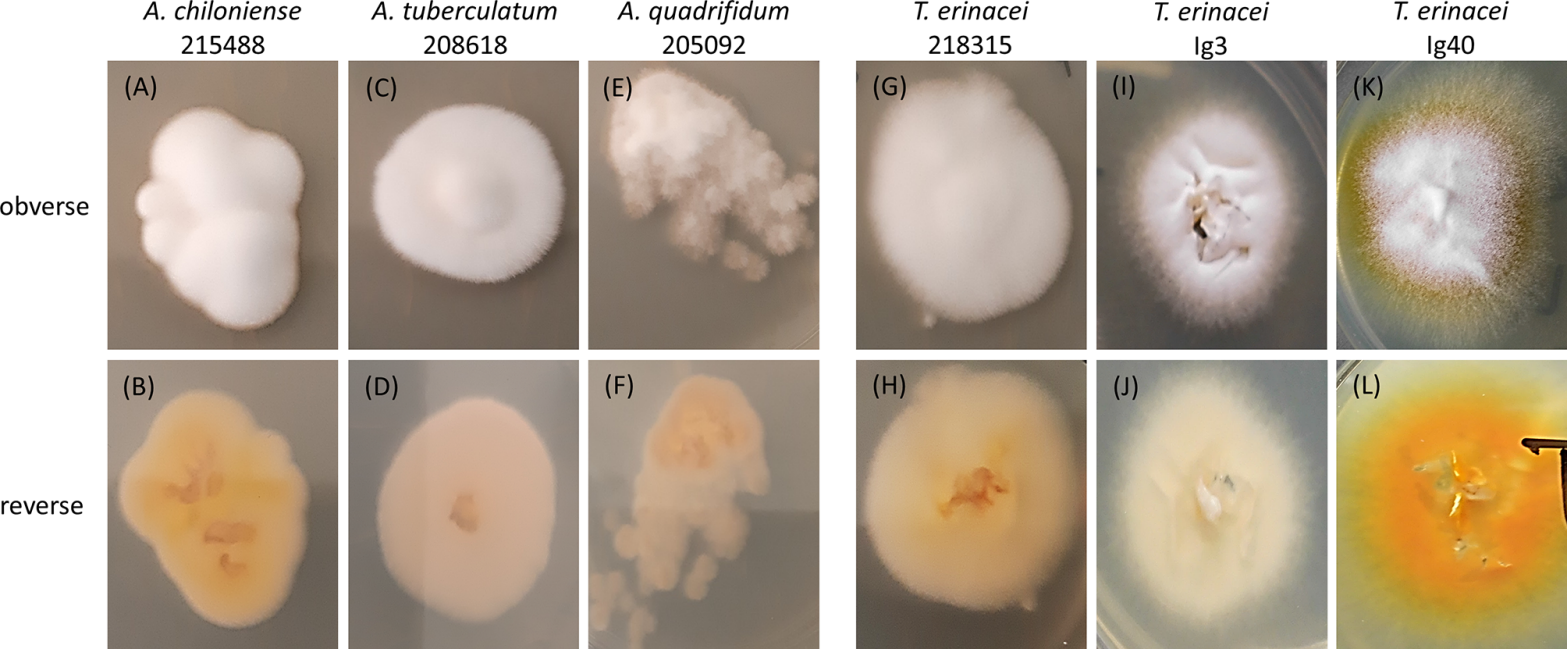
Photographs of the colony morphology of selected *Arthroderma (A.)* spp. **(A–F)** and *Trichophyton (T.) erinacei* cultures **(G–L)** isolated from symptomatic and asymptomatic hedgehogs. Cultures were grown on Sabouraud-Dextrose agar (2%) at 28°C for 3d, species identity was confirmed by sequencing of the ITS region of fungal rDNA. The first column shows the typical morphology of *A. chiloniense* with a white, fluffy obverse **(A)** and a beige to yellow reverse **(B)**. *T. erinacei* 218315 **(G, H)** showed a very similar appearance rendering those two isolates hardly distinguishable solely by the assessment of phenotypic traits. The typical colony morphology of *T. erinacei* is depicted in **(K)** (obverse: white to yellow, granular) and **(L)** (reverse: yellow to bright orange). Intermediate, rather untypical morphologies (fluffy, folded, elevated in the center) with varying color shades from white to cream to beige are seen with other *Arthroderma* spp. and *T. erinacei* isolates.

Clinical specimens of suspected dermatophytoses were routinely sampled according to a modified McKenzie Brush technique (Mackenzie, 1963). After a rough disinfection, the margins of lesions were brushed with sterile tooth brushes and hair/spines and skin scales were transferred to Sabouraud-Dextrose (2%; SDA; Sifin Diagnostics GmbH, Berlin, Germany) or modified dermatophyte agar plates (MDA; Sifin; containing 0.4 mg/ml cycloheximide). SDA and MDA were supplemented with 0.05 mg/ml gentamicin-sulfate, 0.05 mg/ml chlortetracycline and 0.1 mg/ml chloramphenicol). The plates were incubated for one to two weeks at 28°C (in suspicion of *T. verrucosum*: 37°C); selected isolates are shown in **Figure 1**. Species identity of pure subcultures was assessed by macroscopic and microscopic examination, sequencing and/or MALDI-TOF MS measurements (see below).

The herein described dermatophyte isolates of wild hedgehogs (*Erinaceus europaeus)* were obtained after submission of the animals to the clinic showing a poor general health condition or injuries from accidents (asymptomatic and symptomatic for dermatophytoses; sampled in 2018 by the Clinic for Small Mammals, Reptiles and Birds, University of Veterinary Medicine Hannover, Hannover, Germany). Sampling ensued from recently deceased or –if medically indicated- euthanized animals as described above (approval by an animal ethics committee not needed).

### Species Identification by DNA Sequencing and Creation of an ITS-Based Dendrogram

Sequencing of the *internal transcribed spacer* region (ITS) of the rDNA with a subsequent similarity search using the Basic Local Alignment Search Tool (BLASTn; https://blast.ncbi.nlm.nih.gov/Blast) was conducted to confirm species identity.

Therefore, total DNA from pure fungal cultures was extracted using the QIAmp^®^ DNA Mini Kit (Qiagen, Hilden, Germany) according to the manufacturer’s instructions with an additional overnight Proteinase K digestion at 56°C. Amplification of the ITS region by PCR was carried out essentially as described in Sharma et al. (2006) using the universal primers V9G (5′TTACGTCCCTGCCCTTTGTA3′) and LSU266 (5′GCATTCCCAAACAACTCGACTC3′) (Sharma et al., 2006). Sanger sequencing was performed by Microsynth Seqlab GmbH (Goettingen, Germany).

**Figure 2:**
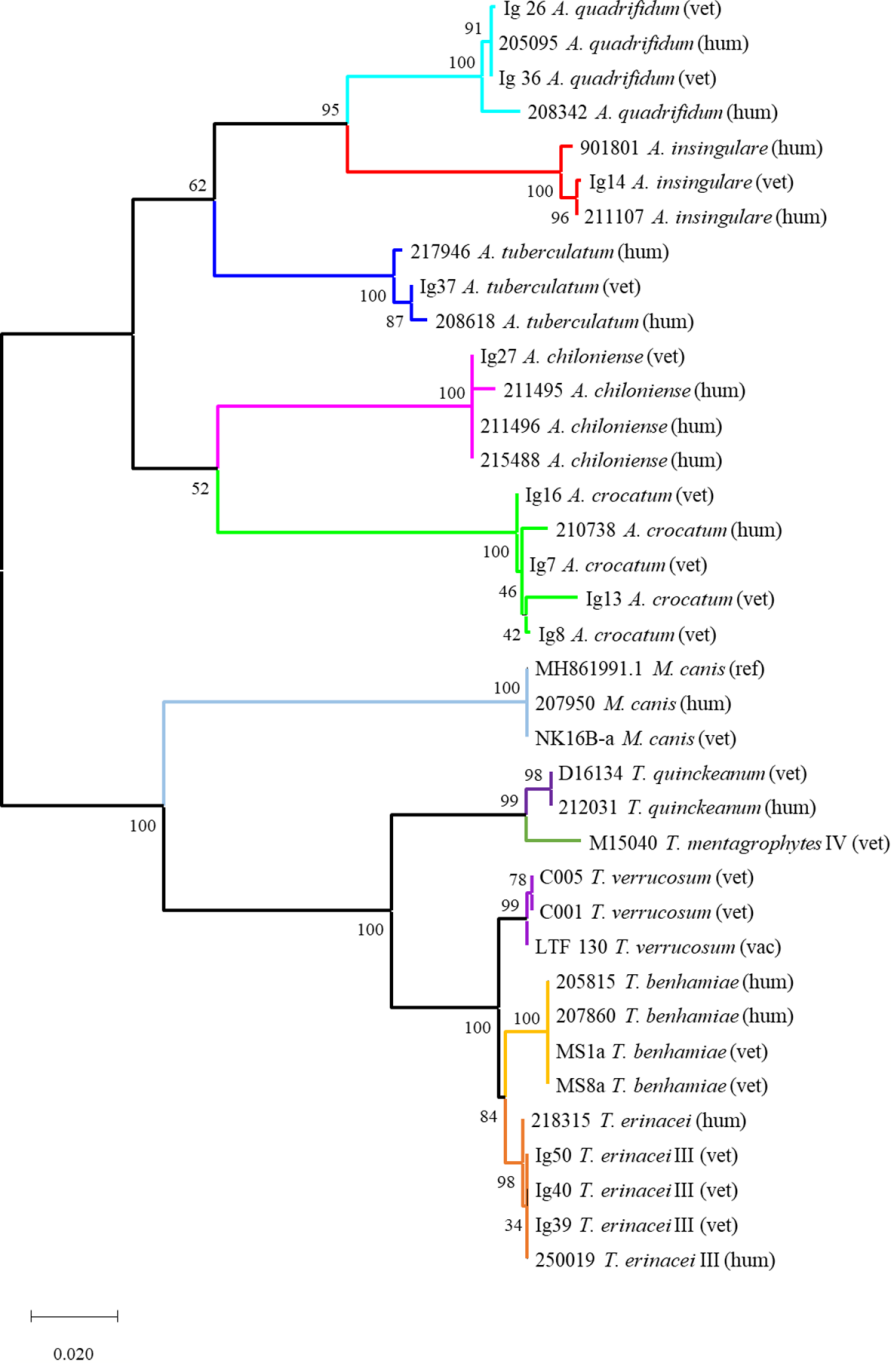
Phylogenetic tree based on fungal rDNA ITS sequences for the investigated dermatophyte isolates (Tamura-Nei-model, Neighbor-Join and BioNJ algorithms, MCL approach, 1000 replicates). The tree is drawn to scale with branch lengths measured in the number of substitutions per site and indicated support values; the analysis involved 37 nucleotide sequences including the dermatophytes isolated during this study and from veterinary routine diagnostics (vet; vac refers to a vaccine strain used in bovine practice), human-derived isolates from the Laboratory of Medical Microbiology (hum) and sequences derived from the NCBI database. Generally, each dermatophyte species forms an own subclade with mostly very robust support; the different origins of isolation (vet vs. hum) are not reflected. The tree comprises two main clades: the upper one contains all geophiles, i.e. all *Arthroderma* spp.; the lower one all isolates of the genera *Trichophyton* and *Microsporum*. The latter form individual subclades in this lower clade; the very closely related species *T. benhamiae* and *T. erinacei* as well as *T. mentagrophytes* and *T. quinckeanum* are separated in distinct subclades.

The obtained sequences were edited using the Chromas 2.6.6 software (Technelysium, South Brisbane, Australia); alignment and phylogenetic analyses were conducted in MEGA X (Kumar et al., 2018) using the Maximum Likelihood method and the Tamura-Nei model (Tamura and Nei, 1993). The percentage of trees in which the associated taxa clustered together is indicated next to the branches (1000 replicates). Values of ≥ 70% represent a robust clade support, values between 70% and 50% are considered moderate and ≤ 50% poor. Initial trees for the heuristic search were obtained automatically by applying Neighbor-Join and BioNJ algorithms to a matrix of pairwise distances estimated using the Maximum Composite Likelihood (MCL) approach, and then selecting the topology with the superior log likelihood value. The tree is drawn to scale with branch lengths measured in the number of substitutions per site and shown in **Figure 2**. The analysis involved 37 nucleotide sequences with a total of 1208 positions in the final dataset including the dermatophytes isolated during this study and during veterinary diagnostics, human-derived isolates from the clinical routine of the Laboratory of Medical Microbiology (with informed patient consent) and sequences derived from the NCBI database.

All fungal isolates obtained during this study are available at the German Collection of Microorganisms and Cell Cultures GmbH (DSMZ, Braunschweig, Germany; see **Table 1** for the detailed assignment of isolates to culture collection identifiers). The corresponding ITS sequences of the *T. erinacei*-isolates were deposited in the NCBI BLASTn database (see **Table 1** for acc. no.), for *Arthroderma* spp., these are in preparation.

**Table 1:** Identification of dermatophytes isolated from skin and spine samples of hedgehogs *(Erinaceus europaeus)* by PCR and sequencing of the *internal transcribed spacer* (ITS) region of fungal rDNA.

Date of Report	Lab-No.	Species^a^	Highest Similarity to NCBI entry (acc. no., culture collection identifier)^b^	Percent identity	Culture Collection Identifier (NCBI acc. no. of ITS sequence)^c^
07/08/2018	250030 (Ig1)	*T. erinacei*	MH860764 CBS 511.73	99.83%	DSM 109030 MN961146
07/08/2018	250031 (Ig3)	*T. erinacei*	MH860764 CBS 511.73	99.83%	DSM 108356 MN961147
04/16/2019	600135 (Ig7)	*A. crocatum*	LR746284 CCF 5207	99.41%	DSM 109743
04/16/2019	250171 (Ig8)	*A. crocatum*	LR746284 CCF 5207	99.85%	DSM 109028
04/16/2019	600136 (Ig13)	*A. crocatum*	LR746284 CCF 5207	98.08%	DSM 109753
01/02/2019	250170 (Ig14)	*A. insingulare*	NR_144885 CBS 521.71	98.86%	DSM 109197
04/16/2019	600137 (Ig16)	*A. crocatum*	LR746284 CCF 5207	99.26%	DSM 109752
01/24/2019	600006 (Ig18)	*T. erinacei*	MH860764 CBS 511.73	99.83%	DSM 109203 MN974534
01/24/2019	600007 (Ig20)	*T. erinacei*	MH860764 CBS 511.73	99.83%	DSM 109202 MN974535
01/24/2019	600008 (Ig21)	*T. erinacei*	MH860764 CBS 511.73	99.83%	DSM 109201 MN974536
01/02/2019	250172 (Ig26)	*A. quadrifidum*	LR746285 CCF 5792	99.84%	DSM 109172
01/02/2019	250173 (Ig27)	*A. chiloniense*	LT992885 CBS 144073	99.89%	DSM 109029
01/02/2019	250169 (Ig36)	*A. quadrifidum*	LR746285 CCF 5792	100.00%	DSM 109171
01/02/2019	250174 (Ig37)	*A. tuberculatum*	NR_077140 CBS 473.77	99.06%	DSM 109027
01/24/2019	600009 (Ig39)	*T. erinacei*	MH860764 CBS 511.73	99.83%	DSM 109200 MN974537
01/24/2019	600010 (Ig40)	*T. erinacei*	MH860764 CBS 511.73	99.83%	DSM 109199 MN974539
01/24/2019	600011(Ig50)	*T. erinacei*	MH860764 CBS 511.73	99.83%	DSM 109198 MN974538

a Species identity was deduced from sequencing the ITS region of fungal rDNA and similarity searches using the Basic Local Alignment Search Tool for nucleotide sequences (BLASTn; NCBI).

^b^Accession numbers (acc. no.) of most similar sequences; CBS – Culture Collection of fungi and yeasts; Utrecht, The Netherlands; CCF, Culture Collection of Fungi; Prague, Czech Republic.

c All isolates of this study were subsequently deposited in the German Collection of Microorganisms and Cell Cultures GmbH (DSMZ, Braunschweig, Germany) and thoroughly sequenced for deposition in the NCBI BLASTn database.

### MALDI-TOF MS Measurements

MALDI-TOF MS analyses were carried out using a MALDI Biotyper™ MBT™ smart instrument (Bruker Daltonik GmbH, Bremen, Germany) and the internal libraries “BDAL” (8468 MSPs, 2969 species; 12/09/2019) and “Filamentous Fungi” (577 MSPs, 180 species; 12/09/2019). Samples for MS measurements were prepared according to the standard operating procedure (SOP) of the manufacturer following a modified extended direct transfer or the extraction sample preparation method (SOP 1867813 “Cultivation and Sample Preparation for Filamentous Fungi”; Bruker Daltonik GmbH) and deposited on a polished steel target (MSP 96, cat. no. 8280800, Bruker Daltonik GmbH). The minor modification consisted of a 2min incubation of fungal material in 70% formic acid in a 1.5ml reaction tube and vigorous pipetting before transferring 1µl of the supernatant to the target (rather than the successive application of “front mycelium” and formic acid to the same target spot). The target was subsequently loaded into the MS instrument and measurements were carried out in linear positive-ion mode within a mass range of 2-20kDa using the MBT_AutoX_FilFungi settings in the FlexControl software (version 3.4.204.10, Bruker Daltonik GmbH). Additional settings included: ion source 1 voltage: 20kV, ion source 2 voltage: 18.3kV, lens: 6kV, linear detector: 2694V. LogScore values (scores) from 0 (no similarity) to 3 (perfect match) were automatically calculated against the entries of the above-mentioned internal libraries. The manufacturer recommends cutoff values of ≥ 1.7 and ≥ 2.0 for a probable/secure identification at genus- and species-level, respectively. Additionally, we considered 5 to 10 of the next best hits in the identification score matching chart given by the software and their corresponding scores for final species determination.

### Creation of Master Spectra (MSP) and a Score-Oriented Distance Dendrogram

**Figure 3:**
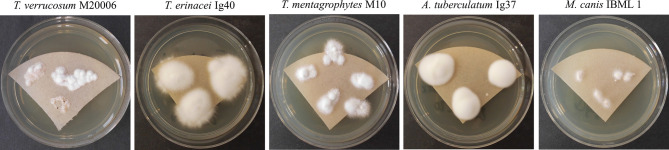
Sabouraud-Dextrose agar (2%) covered with a sterilized filter paper promoted fungal growth successfully and facilitated sampling of merely fungal material for MALDI-TOF MS analysis. Different *Trichophyton (T.), Arthroderma (A.) and Microsporum (M.)* isolates are exemplarily shown after an incubation time of 5d at 28°C (*T. verrucosum:* 37°C). Species identity was confirmed by sequencing of the ITS region of fungal rDNA.

For the creation of own MSPs, fungi were grown on one of the above-mentioned solid agar media covered with a sterilized filter paper (filter circle, Ø 70 mm, type 1573, Schleicher & Schuell, now Whatman; **Figure 3**) or in liquid Sabouraud-2% Dextrose-broth according to SOP 1867813 (section 5.3 “Liquid Cultivation Sample Preparation Procedure”; Bruker Daltonik GmbH). Note that for “Liquid Cultivation”, an incubation no longer than 1-2d in the respective culture broth is strongly recommended by the manufacturer; however, on solid media, dermatophyte growth is considerably slower and more time is usually needed to obtain sufficient fungal material from direct specimens for species identification.

Samples from both growth conditions (solid vs. liquid) were prepared for MS measurements following the extraction sample preparation procedure described in the above-mentioned SOP, i.e. washing fungal material in HPLC-grade water and ethanol and a subsequent formic acid/acetonitrile treatment. After the last centrifugation step, each of at least 12 spots of an MBT Biotarget 96 (cat. no. 1840375; Bruker Daltonik GmbH) was covered with 1µl of this solution. The spots were air dried and finally overlaid with 1µl of saturated α-cyano-4-hydroxy cinnamic acid solution (HCCA matrix; Bruker Daltonik GmbH) for co-crystallization. The bacterial test standard (BTS, cat. no. 8255343; Bruker Daltonik GmbH) provided by the manufacturer was added to the Biotarget 96 as the calibration standard and positive control.

**Figure 4:**
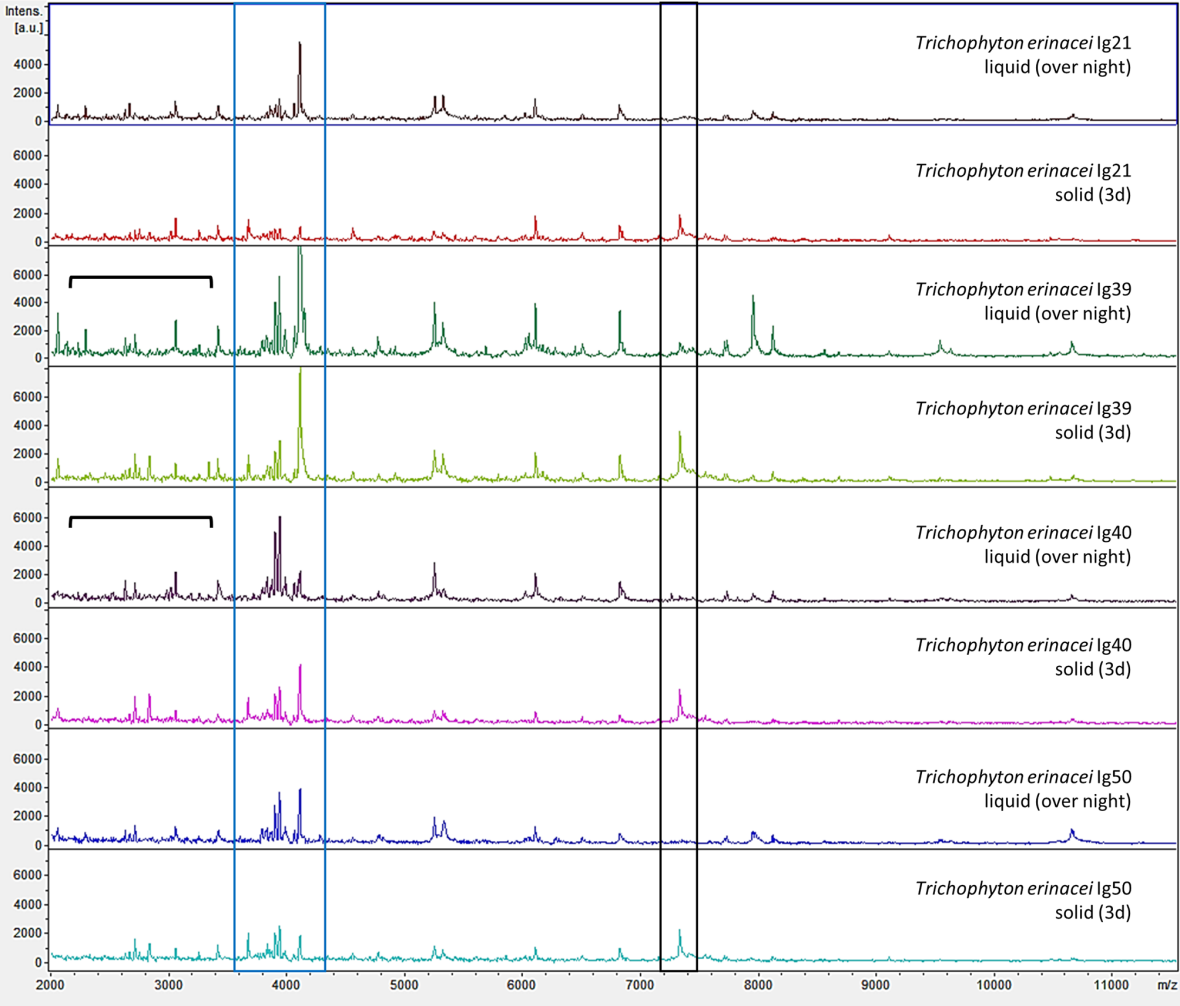
Examples of mass spectrometric profiles (raw spectra) of different *Trichophyton (T.) erinacei*-isolates cultured in liquid Sabouraud-2% Dextrose-broth over-night with gentle rotation at room temperature (liquid, over-night) and on solid agar plates for 3d at 28°C (solid, 3d) are shown. Species identity was confirmed by sequencing of the ITS region of fungal rDNA. Overall, the mass spectrometric profiles were very similar for both cultivation methods. For some isolates grown in liquid media, more and/or more intense peaks were observed in the lower mass range (indicated by the black parenthesis). On the other hand, a peak at around 7300 m/z (black box) was predominantly found in solid cultures. However, a cluster of peaks around 4000 m/z (blue box) seems somewhat species specific for *T. erinacei* since it was found in most examined isolates regardless of the cultivation method.

MS measurements were carried out using the MBT_AutoX_FilFungi settings in the FlexControl software (see above). Each spot was measured twice to obtain at least 24 raw spectra of each sample; selected raw spectra from different isolates and growth conditions are shown in **Figure 4**.

The subsequent quality control of these raw spectra for MSP creation was performed using the FlexAnalysis software (version 3.4.79.0; Bruker Daltonik GmbH) and included baseline correction, smoothing and peak filtering. MSPs were created by combining the remaining raw spectra that fulfilled the quality requirements using the “MSP creation” function of the MBT Compass Explorer software (Bruker Daltonik GmbH). Afterwards, they were scored by the “Start Identification” function (MBT Compass Explorer; Bruker Daltonik GmbH) through comparison to the above-mentioned libraries and own library-entries after MSP creation.

**Figure 5:**
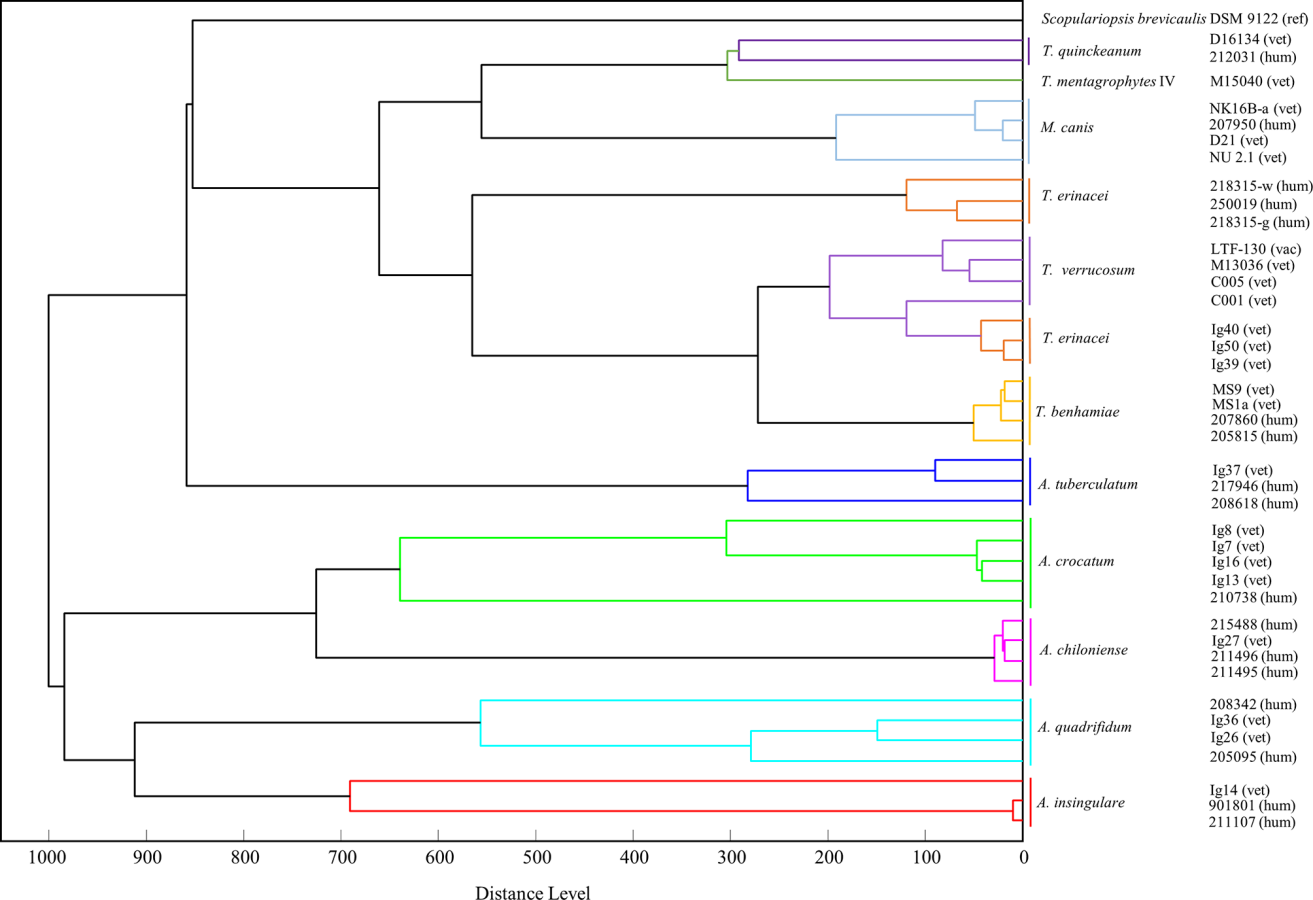
A score-oriented distance dendrogram based on the mass spectrometric data (MSP) was generated to identify similarities and clusters of closely related zoophilic and geophilic dermatophytes (Distance Measure Correlation mode, MBT Compass Explorer, Bruker Daltonik GmbH). Isolates of *Trichophyton (T.) erinacei, Arthroderma (A.)* spp. as well as others from the genera *Trichophyton* and *Microsporum*, respectively, from animal (vet, vac) and human patients (hum) were included (ref - reference, derived from NCBI database). The different species cluster in two main clades: the lower one comprises geophilic isolates only, i.e. *A. insingulare, A. quadrifidum, A. chiloniense* and *A. crocatum*. The isolates of *A. tuberculatum* group into the upper clade but therein in the lowest position, i.e. closest to the other *Arthroderma* spp. The upper clade further comprises the *Trichophyton* spp. and the *M. canis*-isolates (and *S. brevicaulis*). All *T. erinacei, T. verrucosum* and *T. benhamiae-*isolates form one subclade indicating their close relation. Species identity was previously confirmed by sequencing of the ITS region of fungal rDNA.

A score-oriented distance dendrogram based on the mass spectrometric data of MSPs (intensity [arbitrary units]/mass-to-charge-ratio) was generated to identify similarities and clusters of the fungi isolated during this study and during routine human and veterinary diagnostics (Distance Measure Correlation mode, MBT Compass Explorer, Bruker Daltonik GmbH; **Figure 5**).

## Results

### ITS-Based Identification and Phylogenetic Analysis of Dermatophytes

From the cohort of 50 hedgehog-samples of suspected dermatophytoses, 17 samples (34%) gave rise to fungal colonies on SDA or MDA and were subjected to species identification by MALDI-TOF MS and ITS sequencing. None of these cultures were identified using MALDI-TOF MS in the first place (samples were read but not identified; note: in 2018/2019, the above-mentioned internal libraries “BDAL” and “Filamentous Fungi” did not contain MSP of the later identified species). Due to uncharacteristic macro- and micromorphologies as exemplified in **Figure 1**, an unambiguous identification by phenotypic traits was also not possible. Therefore, species identity was analyzed by PCR, ITS sequencing and similarity searches revealing the most common causative agent of hedgehog-dermatophytosis, namely *T. erinacei* (n = 8; 16% of the total sample size), and five different *Arthroderma* spp. (n = 9; 18%) being rare geophiles (*Arthroderma (A.) crocatum*, *A. quadrifidum*, *A. insingulare*, *A. tuberculatum*, *A. chiloniense*; **Table 1**).

A dendrogram based on these ITS sequences was compiled to show similarities between the analyzed dermatophytes (**Figure 2**). Generally, each species formed an own subclade. Different origins of isolation, i.e. human-derived (hum) or from veterinary practice (vet – veterinary; vac - vaccine strain) were not reflected. The tree comprised two main clades: the upper one contained all *Arthroderma* spp., the lower one all isolates of the genera *Trichophyton* and *Microsporum*. The latter formed individual subclades in the lower main clade. *T. benhamiae* and *T. erinacei* as well as *T. mentagrophytes* and *T. quinckeanum* were separated in distinct subclades. Overall, the ITS-based dendrogram is in agreement with reliable identification. However, in the case of species identification with the genus *Arthroderma* this identification relies only on ITS sequences deposited in the NCBI database.

### MALDI-TOF MS Identification of Dermatophytes

Based on the sequencing results, MSPs of all of the above-mentioned 17 cultures were created and deposited in the in-house library to facilitate future identification. We obtained best quality raw spectra from the double-measurement of at least 12 replicates of the same protein extraction (at least 24 raw spectra) rather than triplicate measurements of 8 replicates (as recommended by the manufacturer). More measurements per spot lead to less identical raw spectra that would less likely pass quality control. Moreover, with our method using less measurements per spot, the manufacturer-recommended minimal number of raw spectra for the creation of reliable MSPs (n = 20) was usually obtained.

During further analyses, multiple measurements of cultures gave rise to spectra of varying quality and to variable scores. To find out whether the poorer scores resulted from different culture conditions (solid vs. liquid) or incubation periods (over-night vs. several days), selected dermatophyte isolates were cultured with both methods and measured after different incubation times (**Supplementary Table 1**). The cultivation on solid media was conducted using the above-mentioned filter papers to reduce contamination of the spectra by agar traces. Noteworthy, fresh subcultures were investigated which generally show faster initial growth in comparison to primary cultures of clinical samples. Using the afore-mentioned Bruker libraries only, some of the tested dermatophyte species were not identified correctly, e.g. *T. verrucosum* or *T. benhamiae*, and often with scores at or below the cutoff value for “probable genus identification” recommended by the manufacturer (≤ 1.7). Liquid cultures mostly obtained higher scores compared to time-matched solid cultures (**Supplementary Table 1**). On the other hand, *M. canis* was identified correctly with the internal Bruker libraries after growth of subcultures up to 5d using solid and liquid media (**Supplementary Table 1**). However, *T. verrucosum* IBML C005 was misidentified as *T. erinacei* with scores above 2 using the Bruker database after cultivation on solid medium (not recommended by Bruker; **Supplementary Table 1**).

Applying the in-house library, a “secure genus identification, probable species identification” with scores ≥ 2.0 was usually computed for all cultures of *T. verrucosum*, *T. benhamiae*, *T. erinacei* and *M. canis* grown in liquid and on solid media likewise, provided relatively fresh cultures (up to 5d of growth) were used and time- and cultivation-matched MSPs were included prior (**Supplementary Table 1**). Older cultures were more often attributed with poorer scores or not identified at all although the most elaborate procedure of sample preparation for MALDI-TOF MS measurements (i.e. extraction) was performed (data not shown).

Comparing the three different procedures for sample preparation recommended by the manufacturer (direct transfer vs. extended direct transfer vs. extraction), generally, we found the extraction method for dermatophytes most successful and hence implemented it for all MALDI-TOF MS measurements for daily diagnostics. Direct and extended direct transfer with the addition of formic acid to the sample on the target plate mostly lead to “no peaks” and repeated measurements; the modified extended direct transfer method as described above often yielded correct species identification but sometimes slightly poorer scores than the extraction procedure (data not shown).

Taking a closer look at the raw spectra of some *T. erinacei*-isolates of the current study, liquid cultivation resulted in more and/or more intense peaks, particularly in the lower mass range up to 4000 m/z (**Figure 4**, *T. erinacei* Ig39 (liquid, over-night) and *T. erinacei* Ig40 (liquid, over-night), black parentheses). However, that was not seen uniformly for all tested isolates of this particular species. On the other hand, a cluster of peaks around 4000 m/z seems somewhat species specific since this arrangement was seen in most examined isolates (**Figure 4**, blue box). Interestingly, spectra resulting from cultures grown on solid media exhibited a peak at around 7300 m/z which was not seen in spectra derived from liquid cultures (**Figure 4**, black box).

In summary, growth time and culture condition have an influence on individual raw spectra but not so much on computed scores and species identification if relatively fresh cultures are analyzed.

### Distance Dendrogram Based on MALDI-TOF MS

For similarity and cluster analyses, MSPs were used to generate a score-oriented distance dendrogram that is depicted in **Figure 5**. Some of the current isolates of *T. erinacei* and *Arthroderma* spp. (all marked with “Ig”, **Figure 5**) as well as other closely related *Trichophyton* spp. and *M. canis*-isolates from animal and human patients were included in this analysis. *Scopulariopsis (S.) brevicaulis* was chosen as presumed outgroup. The species cluster in two main groups of which the lower one comprises geophilic isolates only, i.e. *A. insingulare, A. quadrifidum, A. chiloniense* and *A. crocatum*. The isolates of *A. tuberculatum* group into the upper clade but therein in the lowest position, i.e. closest to the other *Arthroderma* spp. Apart from that, the upper clade consists of different *Trichophyton* spp., the *M. canis-*isolates and *S. brevicaulis*. All *T. erinacei, T. verrucosum* and *T. benhamiae-*isolates form one subclade indicating their close relation.

## Discussion

MALDI-TOF MS previously proofed a rapid, accurate and reliable method for the identification of microorganisms in clinical settings and basic research. In the last years, the spectrum of species that are readily identifiable increased substantially due to technical refinements, methodological adaptations and constant extension of reference databases (Cassagne et al., 2016). Here, we can report of a good MALDI-TOF MS performance for the identification of the most common zoophilic dermatophyte *M. canis* (Nenoff et al., 2014b). However, when it comes to less frequent or uncommon causative agents, MALDI-TOF MS still reaches its limits quickly due to incomplete reference data bases (Croxatto et al., 2012; Biswas and Rolain, 2013; Sacheli et al., 2020).

Recollecting from our recent routine laboratory diagnostics, of the zoophilic dermatophytes only *M. canis* was identified reliably with trustworthy scores (n = 10, score ≥ 2.0) and identical next best hits using the provided libraries (BDAL, Filamentous Fungi, Bruker Daltonik GmbH). Supposedly, the high number of deposited spectra of different *M. canis*-isolates (n = 11; 11/20/2020) enables this fast and correct identification. For *T. erinacei*, the most common causative agent of dermatophytoses in hedgehogs, the latter was difficult probably because as few as two reference spectra were only recently introduced to the Bruker libraries.

Within the Bruker MALDI-TOF MS system, the libraries are based on an isolate-specific reference approach in which spectral data are computed from replicates of the same isolate. Different references are not linked and do not influence each other as opposed to the taxonomical group-specific approach pursued by other systems, e.g. Axima@Saramis (Shimadzu/AnagnosTec, Duisburg, Germany) and Vitek MS (bioMérieux, Marcy‐l´étoile, France). Although this isolate-specific approach relies on the correct species identification prior to deposition in the library, it enables the entry of new, customized references immensely, which is a huge advantage in speeding up routine workflows (Cassagne et al., 2016). After creation of MSPs of the above-mentioned *T. erinacei*-isolates and deposition in the in-house library, identification of such samples became a lot more successful. A designated, larger test cohort comprising among others different zoophilic dermatophytes not used for MSP creation will be assessed in the near future to confirm the reliability of the in-house library in a broader context.

Interestingly, although the Bruker databases comprised quite a few spectra of the genus *Arthroderma*, our samples were not identified in the first place; only DNA sequencing provided full account of the different *Arthroderma* spp. Dermatophytoses is often asymptomatic in hedgehogs (66% in our cohort) but if spines are affected and ultimately lost, it may also be lethal and demands special medical attention including the correct identification of disease-causing agents (Weishaupt et al., 2014; Abarca et al., 2017). Moreover, since pet-hedgehogs have increased in popularity, the transmission of *T. erinacei*-infections to humans is also increasingly reported (Abarca et al., 2017; Gebauer et al., 2018; Kargl et al., 2018; Wiegand et al., 2019). The genus *Arthroderma* comprises anthropophilic, zoophilic and geophilic species as well (de Hoog et al., 2017), rendering a zoonotic transmission and human infection also highly likely.

We ascribe the unexpected multitude of dermatophyte species isolated from the hedgehogs in our study to their way of life. However, the high prevalence in this cohort of animals might also be related to their poor health status.

Critical factors for MALDI-TOF MS-based dermatophyte identification include cultural features (media, incubation time) and sample preparation (method, matrix) as well as dermatophyte (species resolution, taxonomy) and mass spectrometer/system characteristics (instrument, library; L’Ollivier and Ranque, 2017).

Concerning the media used, although one might expect different protein expression profiles of dermatophytes grown on different media as a thigmotropic or nutritional response to certain environmental conditions, many studies describe no or negligible effects of different media compositions on the MALDI-TOF-MS identification performance (Theel et al., 2011; L’Ollivier et al., 2013; Bartosch et al., 2018). However, to the best of our knowledge, this is the first study describing the comparison of solid vs. liquid media formulations.

In our study, the usage of liquid media for species identification and/or MSP generation did not prove superior to solid media covered with a filter paper. Spectra obtained from both growth conditions were equally well identified using the in-house library and, hence, the more fastidious liquid culture method was not pursued further. This is mainly due to handling inquiries during sample preparation, e.g. even multiple centrifugation steps did not ensure complete removal of culture media and washing buffer. Also, for some isolates no growth was observed even after a prolonged incubation time. We agree furthermore with our colleagues that 1) contaminations are not as easily identified in liquid cultures and 2) this procedure seems inconvenient to be integrated into a routine workflow for fast diagnostics (Lau et al., 2019; Sacheli et al., 2020). Indeed, for some isolates more and/or more intense peaks/spectrum were obtained which might result in a higher specificity for MSP creation but this was not seen generally. In accordance with others, our study proves that cultures grown on solid media also produce high-quality spectra and good identification scores (Respinis et al., 2014; Sacheli et al., 2020). The filter paper ensured neat sampling of merely fungal material without protraction of agar and proved a fast and cost-effective alternative to other commercially available growth media provided with a membrane, as e.g. ID-fungi plates (IDFP; Conidia, Quincieux, France). Indeed, these plates were described to promote faster growth of filamentous fungi due to an optimized composition and pH (Heireman et al., 2020) but we did not notice any growth difficulties with the isolates tested during our study. Even *T. verrucosum*, which is especially slow growing and demanding (Kane and Smitka, 1978), could be harvested unproblematically already after a few days of cultivation. Nevertheless, IDFP might represent an alternative for otherwise fastidious isolates with special nutritional requirements.

Regarding the incubation time of dermatophyte cultures, in accordance with others we observed better identification scores with younger cultures (over-night in liquid broth or up to 5d on solid media; L’Ollivier et al., 2013). We attribute this to the fact that all entries in the relevant Bruker libraries are established using cultures handled according to the “Liquid Cultivation Procedure”, i.e. only over-night-incubation probably leading to more similar protein expression profiles to younger cultures (pers. comm. with Bruker Daltonik GmbH). The reliable identification after such short incubation times - before characteristic morphological traits of fungi are displayed - is one of the main advantages of MALDI-TOF MS (L’Ollivier et al., 2013). Interestingly, others report of higher identification rates with older cultures (up to 14d of incubation) which might be explained with the production of secondary metabolites that are usually only found in mature cultures (Coulibaly et al., 2011; Packeu et al., 2014). We therefore recommend to include cultures of the relevant species of different incubation times into one’s own library to circumvent these complications.

Both dendrograms, i.e. based on ITS-sequences and on MSP data, support the often-described close relation of dermatophytes altogether and within the different genera (de Hoog et al., 2017).

Two main clades are formed in both trees separating geophilic and zoophilic dermatophyte species almost completely. In the MALDI-TOF MS dendrogram, the majority of the geophilic species cluster in the lower clade; the *A. tuberculatum*-isolates are located directly next to this clade and, in terms of subclades, separated from the zoophilic species. In the ITS-dendrogram, the separation between geophiles and zoophiles is even sharper with both groups forming distinct subclades.

Here, furthermore, each species forms its own subclade; this is also seen in the MALDI-TOF MS dendrogram apart from *T. verrucosum* with one isolate clustering together with the veterinary *T. erinacei*-isolates. However, the nested arrangement of *T. erinacei*, *T. verrucosum* and *T. benhamiae* together in one subcluster in the MSP-dendrogram underlines their close interspecies-relation which is not only seen in other proteome analyses (Bartosch et al, 2018) but also reported in other genomic studies (Gräser et al., 2008; Baumbach et al., 2020). Furthermore, the latter is also supported by the current ITS-tree in which these three species form one common subclade as well.

*T. mentagrophytes* forms a subclade with *T. quinckeanum* in both trees; the latter was only recently separated from the *T. mentagrophytes-*complex and classified as an independent species rather than a variant (Uhrlaß et al., 2018).

Interestingly, their seems to be a close relation to *M. canis* as well since the latter isolates are found in close proximity to *T. mentagrophytes* and *T. quinckeanum* in both trees.

In the MALDI-TOF MS dendrogram, the *T. erinacei*-isolates subcluster according to the host they were isolated from, i.e. all veterinary and all human isolates form distinct subclades. Also, *A. insingulare* and *A. crocatum* show this separation according to the origin of isolation. This phenomenon is not observed in the current ITS-dendrogram and was also not seen in a previous study with *T. benhamiae*-isolates derived from human patients and infected guinea pigs (Baumbach et al., 2020). However, for *T. erinacei* this may be explained with the different geographical sampling sites of these isolates (human: Central Germany; hedgehog: Hannover area).

The overall distribution and organization in common subclades among the *Arthroderma*-isolates is very similar in both dendrograms: *A. chiloniense* and *A. crocatum* form a common subclade as well as *A. insingulare* and *A. quadrifidum*; as mentioned before, the *A. tuberculatum*-isolates are somewhat separated from the other *Arthroderma* spp. Brasch et al. identified a rather remote position for *A. quadrifidum* and, furthermore, quite a distance between the former and *A. insingulare* in another ITS-based phylogenetic analysis but they included only one isolate of each investigated species (Brasch et al., 2019). However, similar to the zoophilic dermatophyte, the interspecific sequence divergence in the genus *Arthroderma* does not exceed 2% (Brasch et al., 2019).

Although *S. brevicaulis* is a mold of the *Microascus* genus, it was grouped into one of the main clades of the MSP-dendrogram and not as a real outgroup. *S. brevicaulis* is regularly isolated from nail infections and, hence, typically associated with mold-related onychomycosis; keratinolytic activities are well reported (Issakainen et al., 2007; Macura and Skóra, 2015). Although the majority of the extracted proteins for MALDI-TOF MS are ribosomal proteins rather than secreted proteases, this similar lifestyle compared to dermatophytes may explain the position of *S. brevicaulis* in the dendrogram.

In conclusion, our results suggest that MALDI-TOF MS is a suitable method for the identification and differentiation of zoophilic dermatophytes provided that the reference library is supplemented with laboratory-relevant species, underrepresented and uncommon taxa, and sufficient isolates per species to circumvent the observed intraspecies diversity and cultivation variations (Theel et al., 2011; Croxatto et al., 2012; Respinis et al., 2013; L’Ollivier and Ranque, 2017).To further improve identification rates, we recommend taking the list of the next best hits from the mass spectrometer identification score matching chart into consideration to conclude secured results. Furthermore, combination with ITS sequencing is advisable in critical cases.

## Data Availability Statement

The datasets presented in this study can be found in online repositories. The names of the repository/repositories and accession number(s) can be found below: https://www.ncbi.nlm.nih.gov/genbank/, MN961146-961147; MN974534-974539.

## Ethics Statement

Ethical review and approval was not required for the animal study because the herein described isolates of wild hedgehogs were obtained from samples taken after submission of the animals to the clinic showing a poor general health condition or injuries after accidents (*Erinaceus europaeus*, asymptomatic and symptomatic; sampled in 2018 by the Clinic for Small Mammals, Reptiles and Birds, University of Veterinary Medicine Hannover, Hannover, Germany). Sampling ensued from recently deceased or –if medically indicated- euthanized animals (approval by an animal ethics committee not needed).

## Author Contributions

C-MB: fungal culture, data analysis, writing of original and revised draft, creation of figures. SM: fungal culture, MALDI-TOF MS measurements, creation of MSPs and score-oriented dendrogram, data analysis. MR: conceptualization of the study, sampling. PN: species identification by PCR and sequencing, supervision and administration. SU: fungal culture, species identification by PCR, sequencing and creation of ITS-based dendrogram, deposition of cultures and sequences in the DSMZ and NCBI BLASTn database, respectively. CB: conceptualization, supervision and administration. WS: conceptualization and experimental design, supervision and administration, optimization of fungal culture, MSP creation, data analysis. All authors contributed to the article and approved the submitted version.

## Funding

Self-funded study. The authors acknowledge support from the German Research Foundation (DFG) and Leipzig University within the program of Open Access Publishing.

## Conflict of Interest

The authors declare that the research was conducted in the absence of any commercial or financial relationships that could be construed as a potential conflict of interest.
