# Supplementary material for: Identification of Zoophilic Dermatophytes Using MALDI-TOF Mass Spectrometry
Source: Front Cell Infect Microbiol. 2021 Apr 28;11:631681. doi: 10.3389/fcimb.2021.631681 (PMC8113951; doi:10.3389/fcimb.2021.631681)
Supplement: Supplementary file 1 [file Table_1.pdf]

Suppl. Tab. 1: Comparison of MALDI-TOF-MS identification performance and scores obtained using the internal Bruker libraries only (BDAL, Filamentous Fungi; 11/20/2020) vs. the in-house library under different cultivation conditions indicated as S (solid, Dermatophyte agar plates covered with sterilized filter paper) vs. L (liquid, Sabouraud- 2% Dextrose-broth) subjected to measurements after different incubation times (1, 3 or 5d, respectively).

Dermatophyte strain	Cultivation time [d]	Cultivation method (S/L)	Bruker identification (BDAL, Fil. Fungi)	Bruker Score (BDAL, Fil. Fungi)[d]	In-house library identification	In-house library score[d]
***T. verrucosum* IBML C005** [a]	1	S[b]	*T. erinacei* 120227 04 ETL	2.020	*T. verrucosum* B002-SAB	2.300
		L[b]	*T. erinacei* 120227 04 ETL	1.920	*T. verrucosum* IBML 20006 d2f3d	2.140
	3	S	*T. erinacei* F29 LLH	1.960	*T. verrucosum* F005-SAB	2.240
		L	*T. erinacei* F29 LLH	1.680	*T. verrucosum* LTF130d2	2.060
	5	S	*T. erinacei* 120227 04 ETL	2.110	*T. verrucosum* IBML 20040d2f3	2.480
		L	*T. erinacei* 120227 04 ETL	1.410	*T. verrucosum* LTF130d2	2.040
***T. erinacei* IBML Ig50** [a]	1	S[b]	*T. erinacei* 120227 04 ETL [c]	2.290	*T. erinacei* IBML Ig3 2dFDAd2	2.340
		L[b]	*T. erinacei* 120227 04 ETL [c]	2.330	*T. erinacei* IBML Ig3f	2.350
	3	S	*T. erinacei* 120227 04 ETL [c]	1.790	*T. erinacei* IBML 1818D9D	2.270
		L	*T. erinacei* 120227 04 ETL [c]	1.970	*T. erinacei* IBML Ig3f	2.030
	5	S	*T. erinacei* 120227 04 ETL [c]	1.860	*T. erinacei* IBML Ig39f	2.340
		L	*T. erinacei* 120227 04 ETL [c]	2.230	*T. erinacei* IBML Ig1bf	2.290

***T. benhamiae* IBML 21023** [a]	1	S[b]	*T. benhamiae* DSM 6916 DSM	1.450	*A. benhamiae* Tier 5a-SAB[f]	2.160
		L[b]	*T. benhamiae* 111116 22 IMD	1.930	*A. benhamiae* Tier 3a-SAB[f]	2.390
	3	S	*A. benhamiae* 24 VML[f]	1.510	*A. benhamiae* 207860-BHI[f]	1.760[e]
		L	*T. benhamiae* 111116 22 IMD	1.590	*A. benhamiae* 205850-BHI[f]	2.210
	5	S	*T. tonsurans* 111201 IMVS	1.250	*A. benhamiae* Tier 12b-SAB[f]	1.720[e]
		L	*T. benhamiae* 111116 22 IMD	1.680	*A. benhamiae* Tier 5a-SAB[f]	2.220
***M. canis* Nk16B-a** [a]	1	S[b]	*M. canis* 111116 20 IMD	2.340	*M. canis* NK16-a-BHI	2.480
		L [b]	*M. canis* 111116 20 IMD	2.240	*M. canis* Ref 207950-SAB	2.560
	3	S	*M. canis* 111116 20 IMD	2.100	*M. canis* IBML KK1 fsab	2.300
		L	*M. canis* 111116 20 IMD	2.310	*M. canis* NK16B-a-BHI	2.300
	5	S	*M. canis* 30 VML	1.870	*M. canis* IBML KK2 d2f3d	1.730[e]
		L	*M. canis* 111116 20 IMD	2.480	*M. canis* IBML KK2 d2f1d	2.500

M. – *Microsporum*, *T.* – *Trichophyton*, *A.*- *Arthroderma*, S – solid (Dermatophyte Agar plates (Sifin) covered with filter paper), L – liquid (Sabouraud-2% Dextrose-broth; Sifin)

[a] All isolates were prepared according to the extraction sample preparation method of the manufacturer (SOP 1867813 “Cultivation and Sample Preparation for Filamentous Fungi”; Bruker Daltonik GmbH).

[b] Investigated fungi were fresh subcultures and not from direct specimens. Fungal growth from direct specimens is generally considerably slower and does usually not result in enough material for species identification after 1d of cultivation only.

[c] These measurements were conducted in 2021 after several updates of the Bruker libraries, the latter now comprise different MSPs of *T. erinacei* enabling reliable species identification. However, in 2018/2019 the libraries did not contain any *T. erinacei*-spectra leading to the result "no organism identification possible" or poor scores.

[d] Measurements obtaining log scores between 1.7 and 2.0 are considered as "probable genus identification" (highlighted in yellow) and ≥ 2.0 as "secure genus, probable species identification" (highlighted in green; results below that score or wrong species identifications are indicated in red) by the manufacturer.

[e] These scores underline our concluding remark that secure species identification may only be achieved if in-house libraries are individually extended by laboratory-relevant species, underrepresented und uncommon taxa and especially sufficient isolates per species to circumvent intraspecies diversity and cultivation variations. For these species, 3/5d-S-cultures were not yet included in the in-house library leading to poorer scores.

[f] *Arthroderma benhamiae* was recently reclassified as *Trichophyton benhamiae* (de Hoog et al., 2017)

Reference

de Hoog, GS, Dukik, K, Monod, M, Packeu, A, Stubbe, D, Hendrickx, M, Kupsch, C, Stielow, JB, Freeke, J, Göker, M, Rezaei-Matehkolaei, A, Mirhendi, H, Gräser, Y. Toward a Novel Multilocus Phylogenetic Taxonomy for the Dermatophytes. Mycopathologia 2017; 182 (1-2):5-31: doi: 10.1007/s11046-016-0073-9
